# Powder Metallurgical Processing and Characterization of Molybdenum Addition to Tungsten Heavy Alloys by Spark Plasma Sintering

**DOI:** 10.3390/ma14195756

**Published:** 2021-10-02

**Authors:** A. Raja Annamalai, A. Muthuchamy, Muthe Srikanth, Senthilnathan Natarajan, Shashank Acharya, Anup Khisti, Chun-Ping Jen

**Affiliations:** 1 Centre for Innovative Manufacturing Research, Vellore Institute of Technology, Vellore 632014, Tamil Nadu, India; raja.annamalai@vit.ac.in (A.R.A.); muthe.srikanth@vit.ac.in (M.S.); senthilnathan.n@vit.ac.in (S.N.); achashash99@gmail.com (S.A.); anupkhisti96@gmail.com (A.K.); 2 Department of Metallurgical and Materials Engineering, NIT Tiruchirappalli, Tiruchirappalli 620015, Tamil Nadu, India; muthuchamy@nitt.edu; 3 School of Dentistry, College of Dental Medicine, Kaohsiung Medical University, Kaohsiung 80708, Taiwan; 4 Department of Mechanical Engineering, Advanced Institute of Manufacturing for High-Tech Innovations, National Chung Cheng University, Chia-Yi 62102, Taiwan

**Keywords:** tungsten heavy alloys, powder metallurgy, spark plasma sintering, materials characteristics

## Abstract

The effect of adding molybdenum to the heavy tungsten alloy of W-Ni-Fe on its material characteristics was examined in the current study. The elemental powders of tungsten, iron, nickel, and molybdenum, with a composition analogous to W-3Fe-7Ni-xMo (x = 0, 22.5, 45, 67.5 wt.%), were fabricated using the spark plasma sintering (SPS) technique at a sintering temperature of 1400 °C and under pressure of 50 MPa. The sintered samples were subjected to microstructural characterization and tested for mechanical strength. The smallest grain size of 9.99 microns was observed for the 45W-45Mo alloy. This alloy also gave the highest tensile and yield strengths of 1140 MPa and 763 MPa, respectively. The hardness increased with the increased addition of molybdenum. The high level of hardness was observed for 67.5Mo with a 10.8% increase in the base alloy’s hardness. The investigation resulted in the alloy of 45W-7Ni-3Fe-45Mo, observed to provide optimum mechanical properties among all the analyzed samples.

## 1. Introduction

Traditional tungsten heavy alloys are two-phase composites with W-rich grains and Ni-Fe or Ni-Cu binder phases. The alloy of W-Ni-Fe exhibits good mechanical properties with a rare combination of strength and ductility [1,2]. The fabrication of these alloys is generally achieved through the powder metallurgy technique, with the W phase being dispersed into the Ni-Fe binder to form a matrix phase. While WHAs (tungsten heavy alloys) possess a relatively high density (>17 g/cc), good strength, high-temperature resistance, and thermal stability are also required. They are superior alternatives to other alloys for traditional defense, space, and nuclear applications [3]. In military applications, they are effectively used as kinetic energy penetrators in replacing depleted uranium [4]. They are also used as counterweights and radiation shields.

Alloying additions, such as rhenium, tantalum, cobalt, molybdenum, and oxide-dispersed tungsten heavy alloys, have been explored in some studies [5,6,7,8]. The liquid-phase sintered WHA with Re and Mo alloying displayed a suitable refinement of tungsten grains by controlling the grain growth and providing adequate strength and hardness. Cobalt alloying improves the strength of the W–matrix interface and provides solid-solution strengthening. The strength of oxide-dispersed heavy alloys depends on the microstructural development [9]. Yttrium oxide, lanthanum oxide, and thorium oxide with WHAs show some improvement in alloy hardness [10]. The criterion for molybdenum addition in tungsten heavy alloys controls the grain size by reducing tungsten dissolution in the matrix phase [11]. In a traditional tungsten heavy alloy, the tungsten forms a solid solution with nickel and iron, leading to particle rearrangement in the sintering process. Nickel is a sound activation agent for tungsten. It improves the sintering kinetics by activating the grain boundary diffusion of tungsten [12,13,14]. Molybdenum forms a eutectic liquid with nickel in the liquid form of W with Ni prematurely, thereby restricting the tungsten’s dissolution in the binder phase [15,16]. It provides grain growth inhibition and solid-solution strengthening of the binder phase. The alloying and processing strategies need to be optimized to attain a suitable performance alloy. Heavy tungsten alloys are usually liquid-phase sintered, in which the low melting elements are melted and dispersed over the matrix phase. The W-Ni-Fe alloys are sintered in the temperature range of 1450 °C to 1500 °C with a dwell period of 2 to 5 h, depending on the sintering technique [17]. A longer sintering time leads to a coarse grain microstructure and deterioration of the alloy’s mechanical properties. Moreover, fabrication requires post-processing techniques, such as aging and swaging, to improve the heavy alloy’s mechanical properties [18,19,20]. At low-temperature solid-phase sintering, obtaining optimal mechanical properties is necessary to obtain a better performance alloy in as-sintered conditions, thus avoiding post-processing techniques. The spark plasma sintering (SPS) technique processes the heavy tungsten alloys [21]. Due to the rapid heating rate in the process, the powder elements are stimulated, and the sintering process is completed in a significantly shorter time than other sintering techniques. Using the SPS technique, the densification of pure tungsten can be achieved at a lower temperature (1200–1450 °C) [22]. The present work focuses on the W-Ni-Fe alloy’s performance with Mo additions using SPS processing at 1400 °C. Molybdenum also possesses a very high melting temperature, good electrical conductivity, outstanding thermal conductivity, corrosion resistance, and a low coefficient of thermal expansion and high hardness [23]. The Mo addition reduces the tungsten composition in the matrix and refines the grain size. The dissolution capacity of Mo in Ni and Fe is higher than that of tungsten [24]. The Ni/Fe ratio in the alloy also affects the material properties by producing different microstructures [25]. If the Ni/Fe ratio is greater than 7:3, the ductility of the material increases, and if it is less, the mechanical characteristics decrease; therefore, a ratio of 7:3 is used to give a stronger yield strength, as investigated by Bose and German. Hence, in this research, the Ni/Fe ratio is maintained at 7:3, and Mo is varied at different ratios with tungsten. Without tungsten, one alloy (90Mo-7Ni-3Fe) was produced and analyzed to determine the difference between tungsten-added alloys and tungsten-less alloys and whether molybdenum may replace tungsten without sacrificing mechanical characteristics.

## 2. Methods and Materials

**Figure 1:**
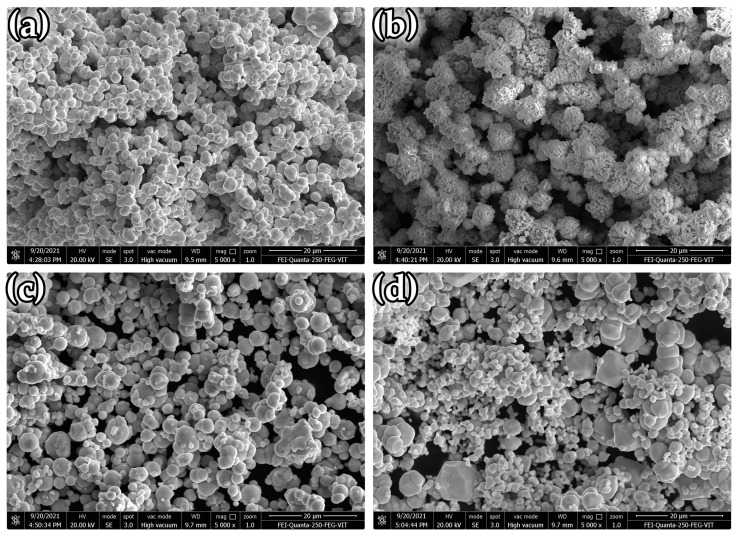
Powder morphology of (**a**) tungsten, (**b**) nickel, (**c**) iron, and (**d**) molybdenum.

Tungsten, molybdenum, nickel, and iron, as the received powders, have an average particle size of 12 μm, <150 μm, <150 μm, and 9 μm, respectively. All of the powders were purchased from Sigma Aldrich, India. Figure 1 shows the SEM morphology of tungsten’s metal powders, molybdenum, nickel, and iron. The powder characteristics are listed in Table 1. The Ni-Fe ratio is maintained at 7:3, and Mo content is varied as 0%, 22.5%, 45%, 67.5% and 90% (composition shown in Table 2). V-Mixer and Ball Mill (without the ball) were used to blend the powders. The given compositions’ consolidation was performed with using the SPS machine (model: DR. Sinter) (Fuji Electronic Industrial Co. Ltd., Tsurugashima Saitama, Japan). Graphite dies with a 30 mm internal diameter were used for the compaction process. A thin foil of graphite was used to distinguish the powder and the punch from the die for the effortless removal of sintered compact. Before sintering, a vacuum was generated in the furnace, which was then filled with argon gas, and the process was repeated three times to achieve a final vacuum of 2 Pa. The powder was sintered at 1400 °C under the uniaxial pressure of 50 MPa, using a heating rate of 100 °C/min. The pressure was maintained over the powder compact during the whole period of the SPS process. The sintering temperature of 1400 °C was maintained in the furnace for five minutes, after which the samples were allowed to cool down in the furnace. The samples with a 30 mm diameter and 7 mm height were produced. The relative sintered density of the samples was determined using the Archimedes principle. Sintered alloys were polished using SiC emery sheets of different grit sizes ranging from 220 to 1200. Diamond paste (6 µm particles) was later used to achieve the sample’s surface mirror finish. Murakami’s reagent (100 mL distilled water, 10 g K_3_Fe(C.N.)_6_, and 10 g KOH) was applied over the polished samples for etching to highlight the grain boundaries. An optical microscope (Zeiss, Oberkochen, Germany) was used to capture micrographs of sintered samples. A scanning electron microscope (SEM, ZEISS EVO 180, Oberkochen, Germany) was used to obtain micro-images with greater magnification.

Energy dispersive X-ray spectrometer (EDS) graphs were obtained simultaneously. The elemental composition of the individual metals was studied using the elemental mapping method. Measurement of the micro-hardness of the sintered samples was conducted with a micro-Vickers hardness tester (Leco, micro-Vickers hardness tester LM248AT, St. Joseph, MI, USA) with an indentation load (0.5 kgf) for a dwell period of ten seconds. Ten readings were taken for each sample by creating indents on the surface at ten random locations. The test result was achieved by calculating the mean of the ten readings. The average grain size was measured using the SEM micrographs taken from the samples [26]. The contiguity (*C_WW_*) was calculated by measuring the number of W–W grain connections (*N_WW_*) and W-Ni-Fe-Mo interfaces (*N_WM_*) using the line-intercept method [27] as given below:

(1) $$C_{WW}=\frac{2N_{WW}}{2N_{WW}+N_{WM}}\;$$

The mechanical properties of UTS (Ultimate Tensile Strength), % elongation, and yield strength were measured using a tensile testing machine (INSTRON 8801, Norwood, MA, USA) following MPIF specifications. The rate of strain used was 3.29 × 10^−4^ s^−1^ (cross-head speed 0.5 mm/min).

## 3. Results and Discussion

### 3.1. Densification Behavior of the Sintered Samples

**Figure 2:**
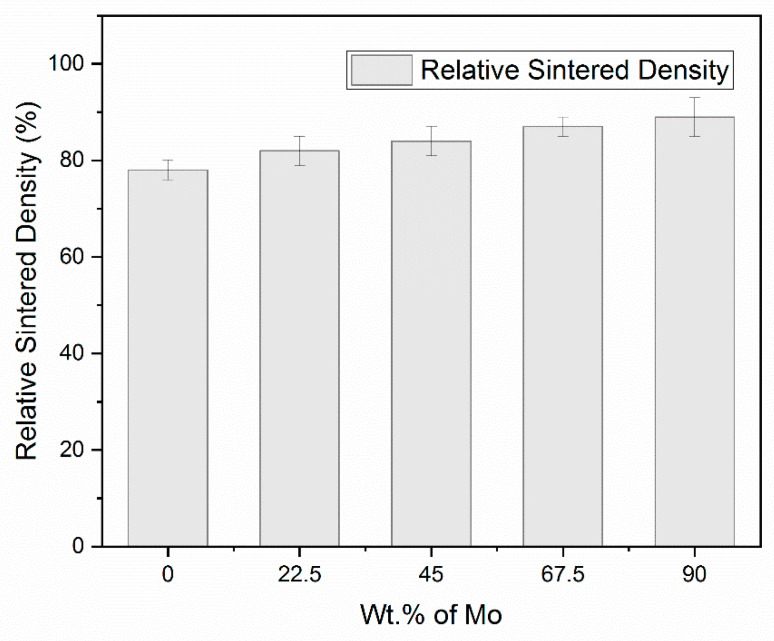
Graphical representation of variation in sintered density with the increase in molybdenum concentration.

The densification behavior showed an increase in the alloy’s relative density with an increase in Mo content, as shown in Figure 2. The process comprised particle rearrangement, dissolution, and precipitation over the binding and diffusion mechanisms at the boundary dividing the matrix phase [28,29]. A maximum relative density of 89.26% was obtained for the 90 wt.% addition of molybdenum. A 15% increase was observed compared to the base alloy. The spark plasma sintering process also aided in improving the sintering kinetics by activating the particles at a high heating rate. The density obtained is in accordance with observations made in other studies on heavy tungsten alloys with molybdenum [30,31].

### 3.2. Microstructure Analysis

**Figure 3:**
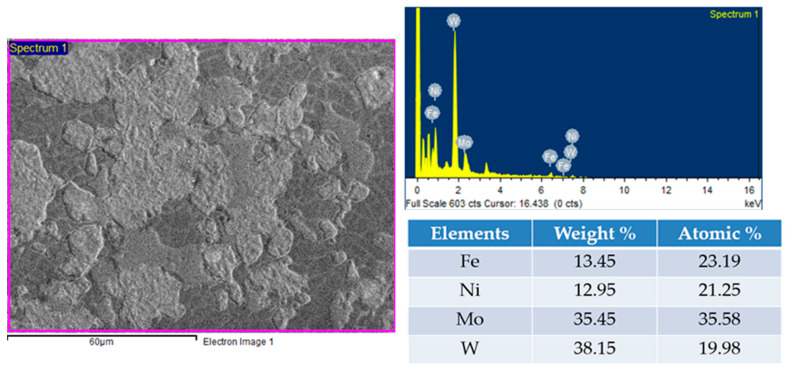
Energy dispersive X-ray spectroscopy of the 45W-7Ni-3Fe-45Mo alloy.

**Figure 4:**
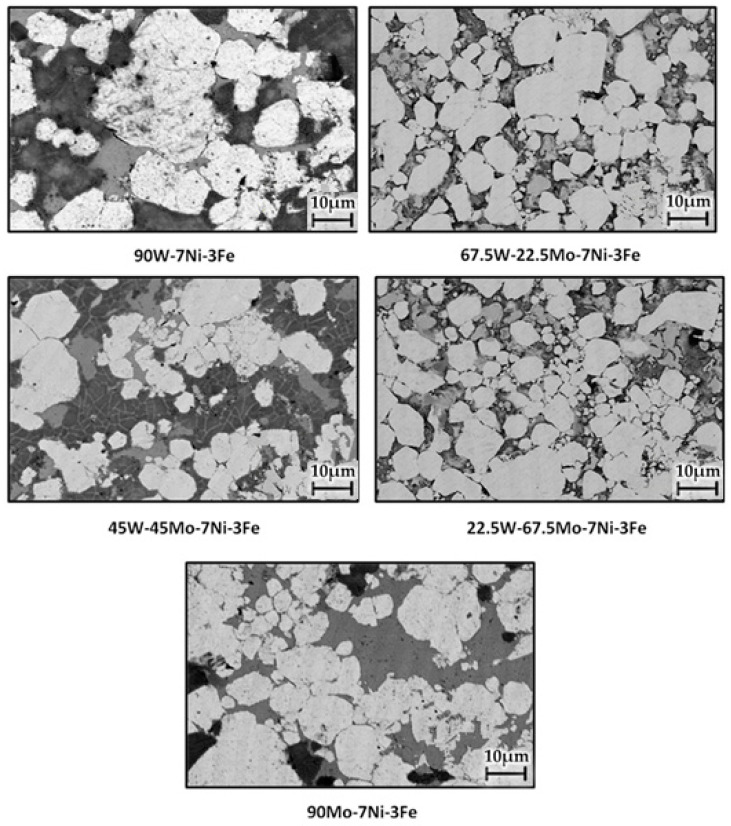
SEM (BSE mode) micrographs of alloys with the increase in molybdenum concentration.

Molybdenum’s addition to WHAs of W-Ni-Fe resulted in changes in the tungsten dissolution in the matrix phase. The contents of the sample were verified in the EDS spectrum, as represented in Figure 3. The microstructure of the sintered samples obtained by varying the proportion of molybdenum is shown in Figure 4. The SEM BSE (backscattered electron) microstructures showed four distinct regions: white tungsten rich phase, light grey molybdenum rich phase, dark grey Ni-Fe rich phase, and pores represented as a thick darker region [32]. The alloy of 90W-7Ni-3Fe showed lesser and non-uniform diffusion of tungsten in the matrix phase with sharp undissolved boundaries visible in the structure. With the addition of molybdenum, more spheroidization and refinement of tungsten grain size were observed.

Mo-added microstructures with a small grain size are shown in Figure 4. The dissolution of tungsten and molybdenum in the Ni-Fe matrix phase evolved with the sintering temperature and time. Molybdenum has very low solubility with tungsten, but it has a greater nickel than W in Ni. At 1100 °C, the Mo solubility in Ni is 25 at.% and that of W in Ni is 16.4 at.% [33]. Thus, the tungsten concentration in the matrix phase was reduced, the microstructure was refined, and the Mo addition improved the solid-solution strengthening of the binder phase.

In all of the alloys, micropores were observed. Porosity is the biggest issue in powder metallurgy-processed components. In all of the alloys, pores are equally distributed throughout the material due to equal heat distribution, but the fraction of porosity is different. The shape of the pores is irregular. Pores act as stress raisers, and they deteriorate the properties of materials.

**Figure 5:**
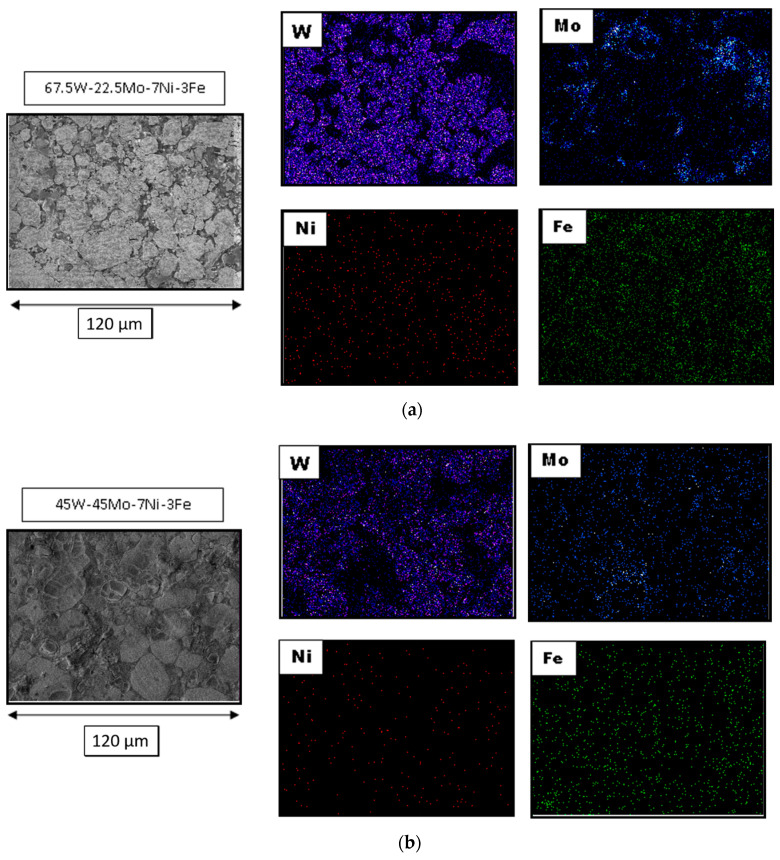
(**a**) Elemental mapping of 67.5W-22.5Mo-7Ni-3Fe, (**b**) elemental mapping of 45W-45Mo-7Ni-3Fe.

The samples’ elemental mapping is shown in Figure 5a,b to verify the distribution of different elemental contents. The variations in grain size, binder volume fraction, and contiguity are all tabulated in Table 3. The grain size of molybdenum-added alloys was observed to be lesser than the base alloy’s size without Mo addition. The smallest grain size of 9.99 microns was obtained for the W-45Mo-7Ni-3Fe alloy. A higher value of 10.22 microns was observed for the 67.5 wt.% Mo-added alloy. This increase is attributed to the change in the matrix volume fraction and the variation in tungsten dissolution in the matrix phase [34]. The spark plasma sintering process also contributed to grain size control. The high heating rate reduced the diffusion process’s total time in the conventional sintering technique and minimized the grain coarsening effect. The contiguity of the W-Ni-Fe alloy was measured to be 0.6093. For molybdenum-added heavy alloys, the contiguity varied from 0.43 to 0.59, which is smaller than the base alloy’s value. Generally, microstructures with minimal contiguity are perceived to provide alloys with good strength and ductility [35]. A high contiguity value represents higher W–W contacts, leading to the phases’ brittleness and thereby reducing the alloy’s ductility. The obtained contiguity values are in accordance with those in the literature on heavy tungsten alloys ranging from 0.4 to 0.6 [36,37]. The matrix volume fraction for the molybdenum-added alloys showed a variation between 32% and 47%. The volume fraction increased with an increase in Mo addition up to 45 wt.%, and there was a corresponding decrease in the microstructure’s contiguity [37].

### 3.3. Mechanical Properties

Heavy tungsten alloys can provide good mechanical properties with a Ni/Fe ratio of 7:3 [25]. The experimental results of tensile strength, yield strength, hardness, and % elongation of the alloys are presented in Table 4. The molybdenum’s ultimate tensile strength added to the tungsten heavy alloys was higher than the alloy without Mo addition. The alloy W-45Mo-7Ni-3Fe exhibited the highest tensile strength of 1140 MPa and a corresponding yield strength of 763 MPa. The sintering and microstructural parameters influence the strength of alloys [38,39]. Rapid heating is followed in the SPS process, resulting in a reduced coarsening of the grains [30]. The refined grain size also contributes to the alloy’s good yield strength based on the Hall–Petch concept [40]. With a reduced grain size, the possible movement of the dislocations created at the grain boundaries restricted the plastic deformation [41]. The lower yield strengths of W-Ni-Fe and Mo-Ni-Fe can be attributed to the corresponding larger grain size and contiguity factors.

The hardness of sintered alloys reflects a growing pattern in the improvement in molybdenum content in alloys. The heavy alloy of W-67.5Mo-7Ni-3Fe contributed to the maximum hardness of 653 Hv, with a significant increase of 10.8% in the hardness (544 Hv) of the heavy tungsten alloy without molybdenum. The Mo addition generally provided a substantial solution strengthening of the binder and improved the cohesion between the tungsten grains and the Ni-Fe-Mo matrix. The ductility of the alloys did not indicate a significant difference with the inclusion of molybdenum. However, the ductility of W-Mo-Ni-Fe alloys was higher than that of the W-Ni-Fe alloy, and a decreasing trend was observed with an increase in molybdenum percentage. This trend is similar to the observations made in other studies on tungsten heavy alloys with Mo addition [30,31]. Mo was highly soluble in the matrix compared to W. As Mo content increased, the solubility of Mo in the matrix increased, and the solubility of W in the matrix decreased, which deteriorated the mechanical properties. When the content of Mo in the alloy was above 45 wt.%, the tensile strength decreased [42].

## 4. Conclusions

The effect of molybdenum alloying with the W-Ni-Fe tungsten heavy alloy was investigated in this research. The results revealed that the yield and tensile strength of W-Ni-Fe-Mo alloys showed a significant improvement compared to those of the W-Ni-Fe alloy. The molybdenum addition provided grain growth inhibition and solid-solution strengthening of the matrix phase, and restricted tungsten phase dissolution. Mo has a higher solubility than that of W. The grain size of the W-Ni-Fe-Mo alloy decreased with an increase in molybdenum content. The alloy W-45Mo-7Ni-3Fe exhibited the highest tensile strength of 1140 MPa and corresponding yield strength of 763 MPa. It was also observed that with the increase in molybdenum in the alloy, the degree of tungsten spheroidization increased. The increase in Mo content also influenced the hardness property, showing a corresponding increasing trend. The maximum hardness was observed for W-67.5Mo-7Ni-3Fe with a 10.8% increase from the base alloy’s hardness. The ultimate tensile strength of the 45% W and 45% Mo alloy was 62% more than that of the parent alloy. 

## Figures and Tables

**Table 1:** Characteristics of W, Mo, Ni, and Fe metal powders.

Powder	W	Mo	Ni	Fe
Particle Size (µm)	12 ± 4	150 ± 5	150 ± 5	9 ± 6
Particle Shape	Irregular	Irregular	Spherical	Spherical
Purity %	99.9	99.9	99.9	99.9
Density (g/cm^3^)	19.28	10.3	8.91	7.86

**Table 2:** Compositions of samples and experimental parameters.

Set No.	Composition	W/Mo Ratio	Compaction Pressure	Heating Rate
1	90W-7Ni-3Fe	100:1	50 MPa	100 °C/min
2	67.5W-22.5Mo-7Ni-3Fe	75:25	50 MPa	100 °C/min
3	45W-45Mo-7Ni-3Fe	50:50	50 MPa	100 °C/min
4	22.5W-67.5Mo-7Ni-3Fe	25:75	50 MPa	100 °C/min
5	90Mo-7Ni-3Fe	1:100	50 MPa	100 °C/min

**Table 3:** Relative sintered density, grain size, and contiguity of all sintered heavy alloys.

S. No.	Specimen Composition (%)	Relative Sintered Density (%)	Grain Size (μm)	Contiguity
1	90W-7Ni-3Fe	78 ± 2	11 ± 5	0.60 ± 0.06
2	67.5W-22.5Mo-7Ni-3Fe	82 ± 3	10. ± 2	0.59 ± 0.08
3	45W-45Mo-7Ni-3Fe	84 ± 3	10 ± 3	0.47 ± 0.1
4	22.5W-67.5Mo-7Ni-3Fe	87 ± 2	10 ± 4	0.43 ± 0.09
5	90Mo-7Ni-3Fe	89 ± 4	10 ± 2	0.57 ± 0.06

**Table 4:** Mechanical properties of all sintered alloys.

Sr. No	Specimen Label	UTS (MPa)	Yield Strength (MPa)	Elongation (%)	Micro-Vickers Hardness (Hv_0.5_)
1	90W-7Ni-3Fe	702 ± 13	442 ± 26	0.17 ± 0.05	544 ± 24
2	67.5W-22.5Mo-7Ni-3Fe	1077 ± 33	722 ± 40	1.51 ± 0.06	595 ± 31
3	45W-45Mo-7Ni-3Fe	1140 ± 26	763 ± 38	0.89 ± 0.08	613 ± 26
4	22.5W-67.5Mo-7Ni-3Fe	815 ± 20	546 ± 40	0.55 ± 0.08	653 ± 19
5	90Mo-7Ni-3Fe	528 ± 54	492 ± 29	0.54 ± 0.04	560 ± 22

## Data Availability

Not applicable.
